# The uptake pattern of 18F-sodium fluoride radioligand in brain tissue after cerebral infarction

**DOI:** 10.1038/s41598-022-26992-4

**Published:** 2022-12-29

**Authors:** Jeong-Min Kim, Reeree Lee, Hae-Bong Jeong, Kwang-Yeol Park, Ju Won Seok

**Affiliations:** 1 Department of Neurology, Seoul National University Hospital, Seoul National University College of Medicine, 101 Daehak-ro, Jongnro-gu, Seoul, Korea; 2 Department of Nuclear Medicine, Chung-Ang University Hospital, Chung-Ang University College of Medicine, 224-1, Heukseok-Dong, Dongjak-Gu, Seoul, 156-755 Korea; 3 Department of Neurology, Chung-Ang University Hospital, Chung-Ang University College of Medicine, Seoul, Korea

**Keywords:** Neuroscience, Neurology

## Abstract

Positron emission tomography with ^18^F-sodium fluoride (NaF) radioligand has been actively investigated in atherosclerosis research because it is known to detect microcalcification activity within atheroma. We studied whether NaF shows any uptake in the brain tissue of patients with acute ischemic stroke. This is a post-hoc analysis of previously reported cerebral atherosclerosis research with positron emission tomography which applied the two radioligands, ^18^F-fluorodeoxyglucose and NaF for the detection of culprit atheroma among 20 acute cerebral infarction patients (mean age = 75.1 ± 9.0 years; 10 women). In this study, we measured the maximum and mean standardized uptake value (SUVmax and SUVmean) of NaF uptake level in the cerebral infarct region between lesions with and without diffusion weighted image (DWI) positivity, indicating acute ischemic cell death. Correlation analysis was performed between NaF uptake levels and imaging and clinical variables, including neurological severity. The NaF uptake levels were significantly higher in DWI positive lesions than in negative lesions (SUVmax: 2.0 [0.60–4.2] versus 0.20 [0.10–0.40], *p* = 0.021 by Mann–Whitney *U* test). The intensity of NaF uptake (SUVmax) was significantly correlated with the initial neurological severity (Spearman's ρ = 0.579, *p*= 0.007) and white blood cell count (Spearman's ρ = 0.626, p *p* 0.003). During ischemic stroke NaF was concentrated in brain tissue undergoing acute cell death and its uptake intensity was correlated with neurological severity, suggesting that NaF could reflect acute ischemic cell death after stroke.

## Introduction

Positron emission tomography (PET) with various radioligands enables the risk stratification of atherosclerosis beyond anatomical imaging^1^. Recently molecular imaging with ^18^F-sodium fluoride (NaF) has been actively investigated in atherosclerosis research because it detects microcalcification activity within atheroma, which is a marker of vulnerable plaque^1^. It exhibited superior efficiency in detecting culprit atheroma among patients with coronary atherosclerosis to ^18^F-fluorodeoxyglucose which shows strong background uptake in the normal myocardium^2^. However, the uptake pattern of NaF in non-calcifying tissues, such as the brain, has been rarely reported. Previous study applying NaF for the detection of culprit carotid atherosclerosis reported that some of the included stroke patients showed strong NaF uptake within brain tissue^3^. The authors speculated that the disruption of cell membrane results in calcium influx and formation of nanoscale calcium phosphate complex which can absorb NaF^3^. Analyzing the uptake pattern of NaF in non-calcifying tissue such as brain would help to understand the NaF uptake mechanism. We investigated the uptake pattern and intensity of NaF within brain parenchyma of stroke patients according to different temporal and spatial relations with cerebral infarction. We also analyzed NaF uptake pattern according to various brain MRI sequences reflecting acute neuronal cell death, hemorrhage and vasogenic edema.

## Methods

### Patient inclusion

This was a post-hoc analysis of cerebral atherosclerosis research with PET, which compared the two radioligands, ^18^F-fluorodeoxyglucose (FDG) and NaF for the detection of culprit atheroma among 20 patients with acute cerebral infarction^4^. The study and its protocol was reviewed and approved by the Institutional Review Board of the Chung-Ang University Hospital (C2015061), and written informed consent was obtained from each patient included in the study according to the Declaration of Helsinki. The inclusion and exclusion criteria and demographic characteristics have been reported from elsewhere^5^. Briefly, acute ischemic stroke patients admitted to Chung-Ang University Hospital with more than 50% carotid stenosis measured by CT angiography were eligible, but patients with active cancer or autoimmune disorder, advanced renal impairment with estimated glomerular filtration rate < 30 mg/mmol, uncontrolled diabetes, or other unstable medical conditions were excluded. All included patients underwent comprehensive stroke etiology evaluation including brain MRI, and neurological status was evaluated using the National Institute of Health Stroke Scale (NIHSS) on admission and modified Rankin scale (mRS) after discharge. Brain MR imaging was performed as described previously with a 3.0-T MR unit (Avanto, Philips, Eindhoven, The Netherlands) including axial and sagittal T1-weighted spin-echo [repetition time (TR) = 450 ms, echo time (TE) = 10 ms, and 5-mm-thick slices with a 1-mm gap], axial T2-weighted fast spin-echo (TR = 3000 ms and TE = 80 ms), axial fluid-attenuated inversion recovery (TR = 9000 ms and TE = 120 ms), susceptibility-weighted imaging (SWI; TR = 22.1 ms, TE = 31.1 ms, and 10-mm-thick slices with 5-mm spacing) and diffusion-weighted imaging (DWI; b = 0 and 1000 s/mm2, TR = 3000 ms, and TE = 74.8 ms) to every patient^6^.

### Positron emission tomography and brain imaging

Whole body PET/CT was performed with a combined scanner (Gemini TF 16, Philips Medical Systems, Cleveland, OH) 60 min after injecting 259–370 MBq (7–10 mCi) of NaF intravenously after eight hours of fasting. PET images were acquired for five min/bed for the head and one min/bed from the skull base to the proximal thigh, immediately after CT scanning (120 kVp, 50 mA). The maximum and mean standardized uptake value (SUV) of the infarcted area were measured at the scan where the infarcted area was the largest by drawing region of interest which can cover most of the infarcted area. The mean SUV of the pons and cerebellum was also obtained from each patient. The mean SUV of the liver, spleen and lumbar vertebrae was determined as described previously^5^.

### Statistical analysis

The clinical and laboratory information was displayed as number of patients (percentage) for categorical variables and median [25th—75th percentiles] for continuous variables. First, we compared the NaF uptake levels according to various brain MRI sequences, including DWI. Since DWI is known to represent early neuronal damage following ischemic stroke by detecting change in the diffusion of water molecules associated with cytotoxic edema^7^, the patients were initially dichotomized into two groups such that the DWI ( +) group included those with DWI-positive lesions and the DWI (-) group, including patients without DWI lesions. We compared the NaF uptake levels in the infarcted regions between the two groups using the Mann–Whitney *U* test. The means of each cerebellar NaF uptake were calculated for each patient, and we compared non-infarcted brain regions, including the pons and cerebellum, as a control. We also compared the mean SUV of NaF in the spleen, liver, and lumbar vertebrae between the two groups. To test whether NaF uptake in the brain could be influenced by hemorrhagic transformation or vasogenic edema due to cerebral infarction, we compared mean NaF uptake between the infarcted regions with and without susceptibility weighted image abnormality, and between the infarcted regions with and without FLAIR abnormality.

Second, Spearman’s correlation analysis was performed between NaF uptake levels and clinical variables, including initial neurological severity and laboratory data, to understand the clinical significance or pathophysiological mechanism of NaF uptake in the brain tissue. The patients were dichotomized into two groups according to the neurological severity and functional independence as mRS 0–2 versus mRS 3- 6 at three months after stroke^8^ and duration between the index stroke and NaF PET investigation. All statistical analyses were performed using SPSS (version 22.0; SPSS Inc., Chicago, IL) and P < 0.05 was regarded as statistically significant.

## Results

A total of 20 stroke patients (median age = 76 years, 10 women) were included in the study and 15 had DWI ( +) lesions, whereas 5 had DWI (-) including four subacute infarction patients and one with transient ischemic attack. Stroke mechanism included 15 atherosclerotic and five cardioembolic stroke cases. One patient passed away after the stroke. The basic demographic and laboratory variables were illustrated in the Table 1.

**Table 1:** Demographic, clinical and imaging variables of the included patients.

Age, years	76 [71–81]
Female patients, n (%)	10 (50)
Hypertension, n (%)	17 (85)
Diabetes mellitus, n (%)	13 (65)
Atrial fibrillation, n (%)	6 (30)
Previous stroke history, n (%)	5 (25)
Osteoporosis, n (%)	5 (25)
Initial NIHSS, mean (SD)	4 [2.5–14.5]
**Infarction size**	
Single lesion within 20 mm	7 (35)
Territorial lesion	11 (55)
Multiple lesions beyond single vascular territory	2 (10)
Time between index stroke and brain MRI, days	5 [3–9.5]
Time between index stroke and PET imaging, days	13.5 [9–20]
**NaF uptake intensity at each organ**	
Infarcted lesions, maximal SUV	1.50 [0.40–3.10]
Infarcted lesions, mean SUV	1.10 [0.35–1.75]
Pons, mean SUV	0.25 [0.20–0.30]
Cerebellum, mean SUV	0.25 [0.20–0.28]
Liver, mean SUV	0.41 [0.39–0.44]
Spleen, mean SUV	0.63 [0.55–0.72]
Lumbar vertebrae, mean SUV	3.26 [2.79–3.51]
Proximal internal carotid artery, mean SUV	1.15 [1.00–1.50]

NIHSS stands for National Institute of Health Stroke Scale; SD, standard deviation; PET, positron emission tomography; NaF, ^18^F-sodium fluoride; SUV, standardized uptake value. Variables are displayed as the number of patients (percentage) or median [25th–75th percentile] as appropriate.

**Figure 1:**
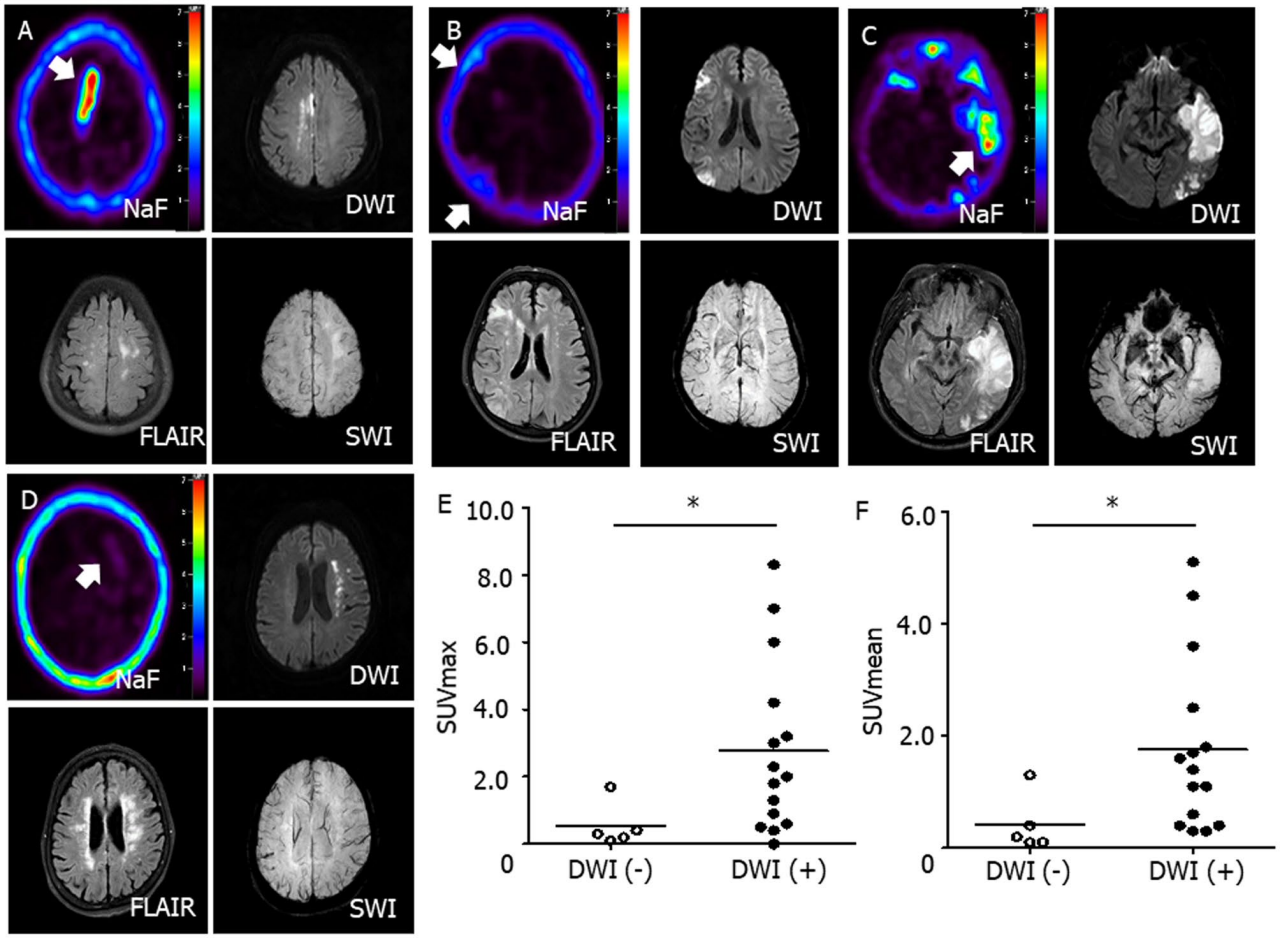
Representative brain imaging and NaF uptake intensity according to infarction lesion. Representative ^18^F-sodium fluoride (NaF) positron emission tomography and brain MR imaging demonstrated that NaF uptake is correlated with acute ischemic lesions from diffusion weighted images from four cerebral infarction patients (**A**–**D**, white arrows). The comparison of NaF uptake intensity between the diffusion-negative and diffusion-positive lesions revealed that both maximum (2.0 [0.60–4.2] versus 0.20 [0.10–0.40], *p* = 0.021, Mann–Whitney *U* test, **E**) and mean (1.4 [0.40–2.5] versus 0.30 [0.20–0.40], p = 0.018, Mann–Whitney *U* test, **F**) standardized uptake values were elevated among the infarction lesions with DWI ( +). DWI stands for diffusion-weighted image; FLAIR, fluid attenuated inversion recovery; SWI, susceptibility-weighted image; SUVmax, maximum standardized uptake value; SUVmean, mean standardized uptake value; *, statistically significant at *p* < 0.05.

**Figure 2:**
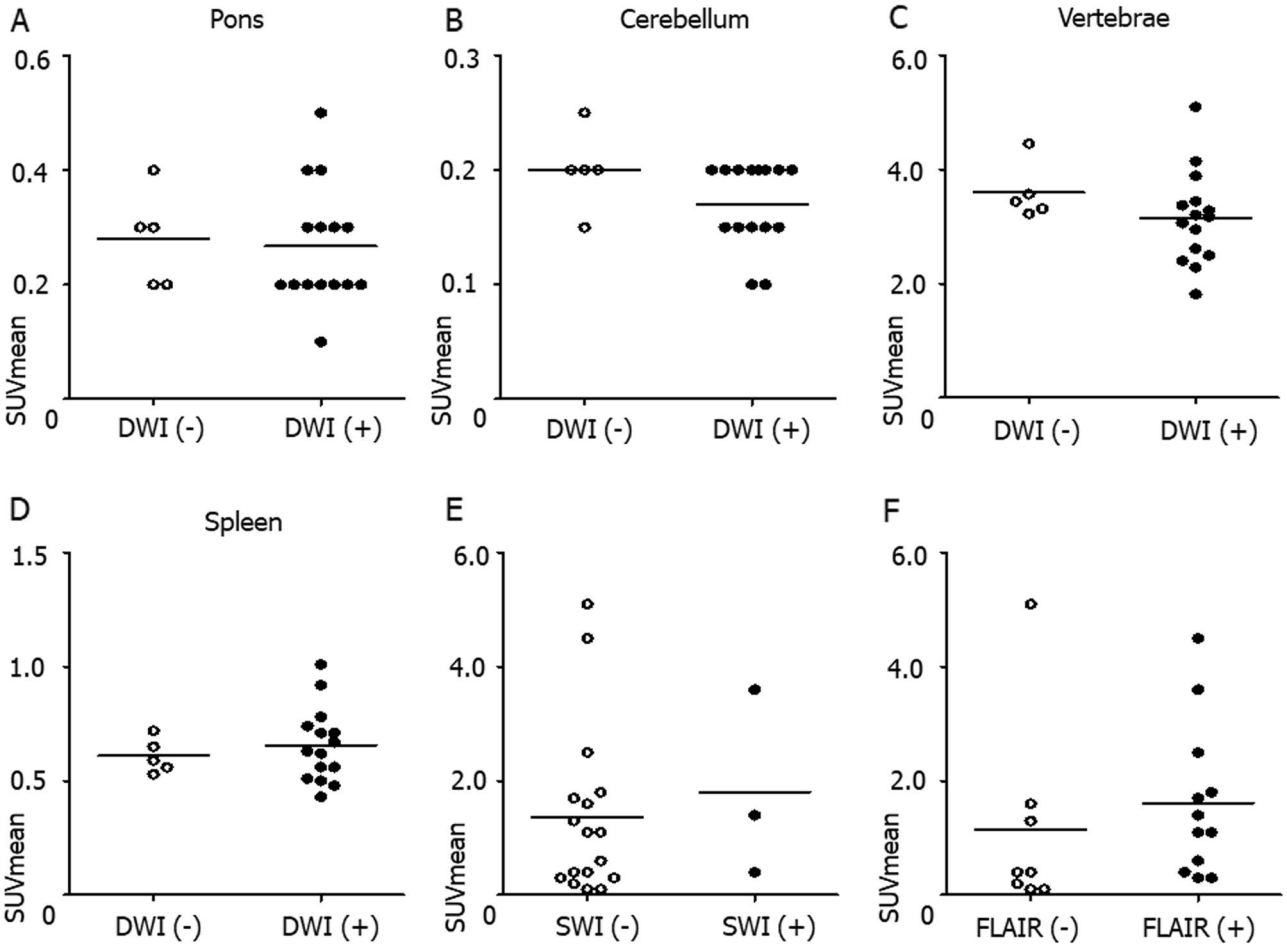
Comparison of NaF uptake according to diverse location and MRI protocol. When comparing NaF uptake in different brain areas and organs between the patients with and without diffusion positive lesions, its uptake intensity was not significantly different in the non-infarcted regions, such as pons (0.20 [0.20–0.30] versus 0.30 [0.20–0.30], *p* = 0.71, Mann–Whitney *U* test, **A**), cerebellum (0.20 [0.20–0.25] versus 0.30 [0.25–0.35], *p* = 0.16, Mann–Whitney *U* test, **B**), lumbar vertebrae (3.17 [2.50–3.38] versus 3.44 [3.31–3.57], *p* = 0.19, Mann–Whitney *U* test, **C**) or spleen (0.63 [0.51–0.71] versus 0.59 [0.53–0.65], *p* = 0.79, Mann–Whitney *U* test, **D**). The NaF uptake was not significantly different in the SWI ( +) lesions (2.50 [1.40–3.60] versus 0.85 [0.30–1.70], *p* = 0.64, Mann–Whitney *U* test, **E**) or FLAIR ( +) lesions (1.25 [0.40–2.50] versus 0.40 [0.15–1.45], *p* = 0.39, Mann–Whitney *U* test, **F**).

Representative images of four stroke patients showed that NaF uptake in the brain is concordant with DWI ( +) lesions on brain MRI, but not with SWI ( +) or FLAIR ( +) lesions (Fig. 1A–D). The maximal (2.0 [0.60–4.2] versus 0.20 [0.10–0.40], *p* = 0.021, Fig. 1E) and mean SUV of NaF (1.4 [0.40–2.5] versus 0.30 [0.20–0.40], *p* = 0.018, Fig. 1F) were significantly higher in the DWI ( +) group than in the DWI (-) group. When comparing NaF uptake in different brain areas and organs between the DWI (-) and DWI ( +) groups, its uptake intensity was not significantly different in the non-infarcted regions, such as pons (0.20 [0.20–0.30] versus 0.30 [0.20–0.30], *p* = 0.71, Fig. 2A), cerebellum (0.20 [0.20–0.25] versus 0.30 [0.25–0.35], *p* = 0.16, Fig. 2B), lumbar vertebrae (3.17 [2.50–3.38] versus 3.44 [3.31–3.57], *p* = 0.19, Fig. 2C) or spleen (0.63 [0.51–0.71] versus 0.59 [0.53–0.65], *p* = 0.79, Fig. 2D). The NaF uptake was not significantly different in the SWI ( +) lesions (2.50 [1.40–3.60] versus 0.85 [0.30–1.70], *p* = 0.64, Fig. 2E) or FLAIR ( +) lesions (1.25 [0.40–2.50] versus 0.40 [0.15–1.45], *p* = 0.39, Fig. 2F).

**Figure 3:**
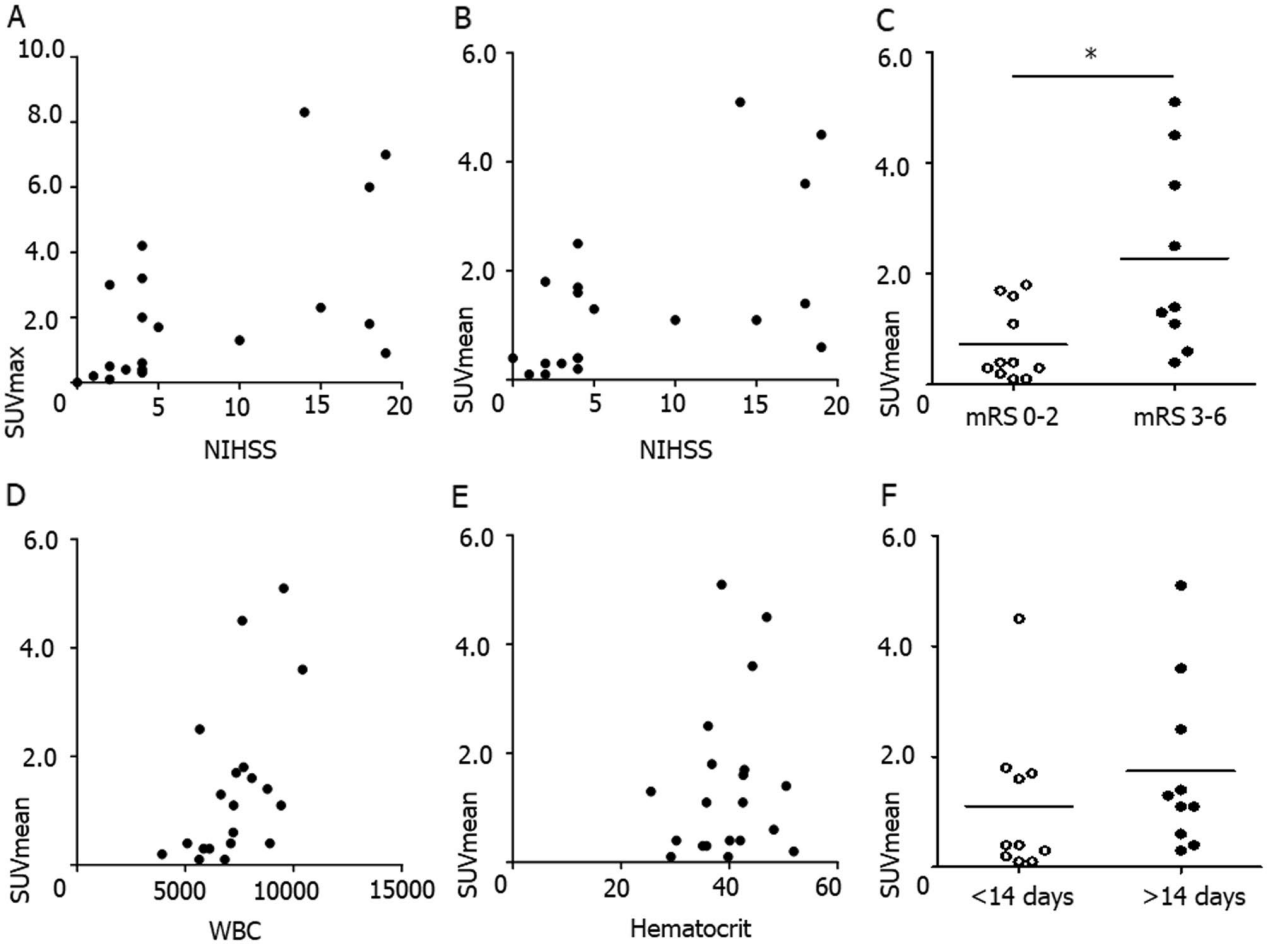
The relationship between NaF uptake and neurological outcome. The correlation analysis between NaF uptake and neurological severity showed that both SUVmax (Spearman’s ρ = 0.579, *p* = 0.007, **A**) and SUVmean (Spearman’s ρ = 0.630, *p* = 0.003, **B**) were significantly correlated with the National Institute of Health Stroke Scale (NIHSS) at admission. The NaF uptake was significantly lower among the patients with modified Rankin scale (mRS) between 0 and 2 than those with mRS greater than 2 at 3 months after stroke (0.35 [0.15–1.65] versus 1.35 [0.50–4.05], *p* = 0.030, Mann–Whitney *U* test, **C**). The uptake intensity of NaF was significantly correlated with white blood cell count at admission (Spearman’s ρ = 0.626, *p* = 0.003, **D**), but not with hematocrit (Spearman’s ρ = 0.259, *p* = 0.269, **E**). When the patients were dichotomized according to the imaging taken after index stroke, the uptake intensity of NaF was not significantly different between the two groups (0.55 [0.15–3.15] versus 1.75 [1.25–5.10], *p* = 0.35, Mann–Whitney *U* test, **F**).

The correlation analysis between NaF uptake and neurological severity after stroke demonstrated that both SUVmax (Spearman’s ρ = 0.579, *p* = 0.007, Fig. 3A) and SUVmean (Spearman’s ρ = 0.630, *p* = 0.003, Fig. 3B) were significantly correlated with the NIHSS at admission. The NaF uptake was significantly lower among the patients with mRS between 0 and 2 than those with mRS > 2 at three months after discharge (0.35 [0.15–1.65] versus 1.35 [0.50–4.05], *p* = 0.030, Fig. 3C). The uptake intensity of NaF was significantly correlated with white blood cell count at admission (Spearman’s ρ = 0.626, *p* = 0.003, Fig. 3D), but not with hematocrit (Spearman’s ρ = 0.259, *p* = 0.27, Fig. 3E). When the patients were dichotomized according to the temporal duration of PET/CT imaging after stroke at 14 days, the uptake intensity of NaF was not significantly different between the two groups (0.55 [0.15–3.15] versus 1.75 [1.25–5.10], *p* = 0.35, Fig. 3F).

## Discussion

When NaF PET was applied to stroke patients, NaF radioligand was localized in the brain parenchyma where acute ischemic cell death was prevalent. Interestingly, hemorrhagic transformation or vasogenic edema following cerebral infarction did not affect NaF infiltration within the brain. The uptake intensity of NaF was proportional to neurological severity and systemic inflammatory response, suggesting a biomarker potential reflecting acute neuronal injury due to ischemia.

It is noteworthy that NaF uptake was detected in neuronal tissue which rarely undergoes calcification. The utility of NaF has been focused on the detection of metastatic cancer invasion of the skeletal system because it exchanges fluoride ions with hydroxyl ions from hydroxyapatite within bone crystals^9^. Recently, it has been actively applied in vulnerable atherosclerotic plaque detection or cardiac valvular calcification considering similar deposition of hydroxyapatite within calcifying atheroma^3^. A comparative study on coronary atherosclerosis showed that NaF can be superior to FDG because it can detect microcalcification which is a marker of unstable atheroma^2^. The application of NaF to carotid atherosclerosis revealed conflicting results according to the included subjects^3,10^. We observed that NaF uptake was higher in the carotid atheroma with severe calcification, but its uptake also tended to be elevated in the atheroma with a necrotic core^10^. Another studies also reported intense NaF uptake in infarcted brain tissue from brain CT^11^. Since hydroxyl ions are a major constituent of free radicals during necrosis, the active ion exchange with fluoride ions could be a plausible explanation. More in vitro and in vivo studies can elucidate the exact mechanism of NaF uptake in infarcted brain tissue.

The selective uptake of NaF toward acute ischemic cell death and the correlation between its uptake intensity and neurological severity suggest that it can reflect acute neuronal injury following stroke. Uptake of bone metabolism markers had been observed in tissue necrosis including myocardial infarction^3^. This could help to detect or quantify the intensity of neuronal injury following ischemia, which can be used as an imaging biomarker in clinical trials of neuroprotective agents. However, this study was conducted as a post-hoc analysis with small number of included patients. We could not perform histological analysis of the brain with intense NaF uptake, which may reveal the exact mechanism of NaF uptake in neural tissue. Whether NaF can provide prognostic information for patients with stroke warrant future studies.

## Data Availability

The data that support the findings of this study are available from the corresponding author upon reasonable request.
